# Army and Navy Notes

**Published:** 1898-09

**Authors:** 


### ARMY AND NAVY NOTES.

THERE is a movement on foot to have assistant surgeons in the navy placed in the rank of lieutenants of the junior grade, which will not only give them a better official standing, but will elevate them socially on ship. As it is now they mess with the petty officers and ensigns, when as a matter of right, justice and position they should be on equal footing with the officers of the line.

They are now on a level with cadets who are just finishing their educations. The qualifications necessary to secure the position of assistant surgeon are such as to entitle that officer and his corps to some consideration. The navy of the United States is a great institution, but it is sadly lacking in discrimination in some things and this is one of them.

SURGEON-GENERAL STERNBERG will, according to a Washington dispatch of August 17, 1898, send out a scientific commission to investigate the causes of typhoid fever in the army camps and report upon a method to prevent such occurrences in the future. The commission will consist of Major Walter Reed, U. S. A.; Major Victor C. Vaughn, division surgeon of volunteers, of the University of Michigan and Major Edward O. Shakespeare, brigade surgeon, U. S. V. The latter, it will be remembered, made an investigation of cholera for the government a few years ago.

---

SINCE our August edition went to press the following-named Buffalo physicians have been appointed acting assistant-surgeons U. S. Army: Drs. J. H. Grant, J. N. Goltra, Julius Ullman, George W. Pattison, John E. Bacon, Harold W. Cowper, H. H. Bradley, Charles W. Farr, Nelson W. Wilson, W. T. Tanner and Charles A. Andrews. Of these Dr. Ullman has been ordered to Chickamauga Park, Dr. Pattison to Fort Myer, Va., Dis. Goltra and Farr to Porto Rico, Dr. Grant to Fort McPherson, Ga., Drs. Bacon and Bradley to Santiago. We are not advised as to the assignments of the others. The surgeon-general makes contracts with these physicians to terminate at the pleasure of the government at from $100 to $150 a month. Dr. Wm. H. Heath has, it is announced, been appointed brigade surgeon with the rank of major.

---

COLONEL WILLIAM H. FORWOOD, assistant surgeon-general United States Army, has been placed in charge of the hospital service at Montauk Point. Colonel Forwood appointed Dr. Ira C. Brown, of Buffalo, as executive officer of the Camp Wikoff general hospital, and on Friday, August 19, 1898, Dr. Brown received a commission as major in the volunteer service, in recognition of his services in the aforementioned capacity. Colonel Forwood recommended his promotion to the War Department and so did General Young The result was Dr. Brown's commission, which dates from August 11th.

Major Brown entered the service on July 21st as acting assistant surgeon United States Army and proceeded to Tampa with the understanding that he was going to Cuba, but the necessity for surgeons at Tampa held him there. He was assigned to duty with the cavalry brigade, Fifth Army Corps, and when they broke camp he was transferred to the 6th Cavalry, remaining with that regiment until it reached Montauk. Major Brown is a graduate of the medical school of the University of Buffalo and his home is in this city.

ANOTHER jaundiced exhibition of modern literature within the past month was the publication in Harper's Weekly of a front page cartoon, representing the deck of the Concho, on which were stretched various emaciated and fever-parched forms attired in the rags of the United States Army. Overhead floated the stars and stripes and a flock of foul vultures. Beside the forms on the deck stood Uncle Sam deep in meditation; kneeling at his feet with the regulation outstretched arms which one sees always in Delsarte's representations of the virgin Grecian girl begging for mercy, is Columbia; and she is saying to Uncle Sam that if he does not fix the responsibility for the horror the people will. The whole thing is the product of a war-distorted imagination and the work of a pencil blunted by the license of cartoonery. The picture, its conception and its publication is unworthy of Harper's Weekly, which according to the sub-title is "A Journal of Civilisation."

---

THE JOURNAL believes Buffalo one of the most healthful spots on the map. Believing so and believing with all good people that the soldiers should be treated in a manner which will result in the greatest good to the greatest number, we suggest that the surgeon-general designate Buffalo and its vicinity as a recuperating station for the soldier boys who have come back from the war ill from exposure to the sun and the rains of Cuba and the forced marches attendant upon the capture of Santiago.

---

THE removal of typhoid fever patients from the field hospitals to general hospitals in large cities is, in our view, not to be commended. Experience has shown that tent hospitals afford the best facilities for the treatment of soldiers suffering from so-called camp fever—malarial, typhoid and the like. In them there is perfect ventilation without a draught, the air is constantly changing night and day, sifting through the canvas and always remaining pure. And this is a most important factor in the care of such patients. To move them even to the environments of comfortable homes is dangerous, prompted though such proposal be by the kindest motives. A soldier sick with fever should be treated during the activity of the malady in a tent; after convalescence is fully established he may be sent home or to a city hospital to complete the recovery.

---

MISS HELEN GOULD, who has been very properly called "the good American girl," has added to her numerous contributions to charity by handing over $25,000 for the relief of the soldiers at Montauk Point. Miss Gould's charity is unostentatious and kindly. She was left an immense fortune and she is putting it where it will do the most good.

---

TYPHOID fever is increasing among the soldiers at Montauk Point. On August 22d there were 225 cases under treatment and a large number of suspects.

---

IT IS hard to believe the published reports regarding the refusal of the army officials to permit the Red Cross to install water distilling plants at Montauk Point, even at its own expense.

The need of pure water is apparent at this point, and medical opinion is unquestioned that if a proper water supply had been provided before the advent of the soldiers, the crushing out of typhoid would have been comparatively easy.

---

MAJOR H. C. BOWEN, surgeon of the second Massachusetts Volunteers is reported to have died at Santiago, August 13, 1898, of typhoid fever.

---

ACCORDING to official and semi-official reports, which are still subject to revision and correction, the number of officers and men of the army killed in action since the outbreak of hostilities against Spain has been 282 and the number wounded, 1,496, making a total of 1,778. It is probable that additions will be made to the list of killed by subsequent reports. Of course, the casualties occurred principally in the fighting around Santiago, but even there the number was not excessively large, considering the stubbornness of the defense, and the fact that infantry, inadequately supported by artillery, was led against an enemy strongly entrenched. Most of the fighting was with small arms, and few men, in the American army at least, were killed or wounded by shells.

